# Total Talar Replacement With Custom-Made Vitallium Prosthesis for Talar Avascular Necrosis

**DOI:** 10.3389/fbioe.2022.916334

**Published:** 2022-05-20

**Authors:** Wenbin Luo, Hanyang Zhang, Qing Han, Zhaoyan Li, Zhuan Zhong, Guoliang Jia, Yuxuan Liu, Fei Chang, Jincheng Wang

**Affiliations:** Department of Orthopedics, The Second Hospital of Jilin University, Changchun, China

**Keywords:** total talar replacement, 3D printing, talar avascular necrosis, Interface structure design, bionic prosthesis Interface

## Abstract

**Background:** The current study investigated the application of three-dimensional (3D) printing technology in the treatment of talar avascular necrosis (TAN). Custom-made Vitallium talar prostheses were designed and generated *via* 3D printing. We hypothesized that these talar prostheses would facilitate more stable positioning, better ergonomically fit the ankle joint surfaces, and promote favorable long-term prognoses.

**Material and Methods:** Computed tomography scans of both ankle joints were acquired from three patients diagnosed with TAN. The talar on the unaffected side was used as the design blueprint. Hence, with the aid of 3D printing technology a customized talar prosthesis made from a novel Vitallium alloy could be manufactured for each individual patient.

**Results:** In all three cases there were no signs of prosthesis loosening or substantial degenerative change in the surrounding area of the joint, but small osteophytes were observed on the tibial side and navicular side. No chronic infection or other prosthesis-related complications were observed in any of the patients. All three were able to walk without pain at the most recent follow-up.

**Conclusion:** With the aid of 3D printing and a novel Vitallium alloy, total talar replacement achieved encouraging results in 3/3 patients. All patients were satisfied with their joint function, and were able to return to their daily activities without limitations. Although more cases and longer-term follow-up periods are required, the success rate reported herein is encouraging.

## Background

The talus is a bone with five articulating surfaces, and nearly 60% of its surface is covered with articular cartilage. It plays a vital role in the daily functioning of the ankle joint. (Lee et al., 2020) Because the majority of the talus’ surfaces are in seamless contact with surrounding bones, the blood supply provided by its remaining bone surface is very fragile and extremely sensitive to injury and other systemic processes that impede blood flow. (Gelberman and Mortensen, 1983) Therefore, talar avascular necrosis (TAN) has been a great challenge for foot and ankle specialists. Treatments for end-stage diseases causing TAN such as advanced ankle arthritis and ankle osteomyelitis remain limited. Treatment options include talus resection, total ankle arthrodesis, total ankle arthroplasty, and below-knee amputation. (Parekh and Kadakia, 2021) Generally ankle joint fusion surgery can provide a stable joint for daily activities, with acceptable follow-up results. In patients with bilaterally affected ankle joints however, ankle arthrodesis in both joints often leads to deterioration of joint function in association with activities such as climbing stairs, getting out of a chair, walking on uneven surfaces, and running wherein flexion and extension of the ankle joints are needed, resulting in poor long-term follow-up results. (Hintermann et al., 2009; Bussewitz et al., 2014; Schuberth et al., 2020) Fusion surgery is also associated with long recovery times, and a proportion of patients exhibit compromised bone healing. (Fragomen et al., 2012) Total ankle replacement (TAR) is another viable treatment option for such ankle diseases, but unfortunately complications of TAR such as component loosening, subsidence, and collapse limit long-term prognoses. Hence, more optimized options should be considered.

Total talar replacement (TTR) has recently received increased attention from foot and ankle specialists as an alternative treatment method in select cases. Since the first reported case in 2010, (Tsukamoto et al., 2010) TTR has achieved satisfactory outcomes in the treatment of talus necrosis and severe crush fracture. (Angthong, 2014; Taniguchi and Tanaka, 2019) Due to the unique anatomic form of the talus, the vital element for a successful prosthetic TTR is the congruency of the custom-made implant with the adjacent joints. (Mu et al., 2021) Despite the rapid development of TTR prostheses however, full reconstruction of the anatomic features of a native talus—which would optimally stabilize and maintain normal ankle function—remains a challenge. (Ando et al., 2016) Three-dimensional (3D) printing technology integrated with computed tomography (CT) imaging and computer-aided design has now been widely used in surgical planning and custom-made implant design, to achieve anatomic accuracy in talar prosthetic design and production. (Akoh et al., 2020; Mu et al., 2021) 3D printing has shown tremendous potential for the design and production of refined structures with irregular shapes and free surfaces such as the talar body. Thus, the utilization of 3D printing technology in conjunction with TTR may be a promising way for surgeons to provide more personalized, anatomically accurate, and above all more durable surgical solutions with better long-term prognoses.

The current study investigated a prosthetic design and production process incorporating a combination of computer-aided design and 3D printing technology. Using the process, three patients with idiopathic talar necrosis were treated *via* TTR with a Vitallium prosthesis. We hypothesized that these 3D-printed prostheses would facilitate more stable positioning, better ergonomically fit the ankle joint surfaces, and promote favorable long-term prognoses.

## Material and Methods

### Ethics Approval and Consent to Participate

This study was conducted in accordance with the principles outlined in the Declaration of Helsinki, and was approved by the Ethics Committee of the Second Hospital of Jilin University, China. Written informed consent to participate was obtained from all patients involved in the study. Patient data were kept anonymous to ensure confidentiality and privacy.

### Patient Characteristics

**FIGURE 1:**
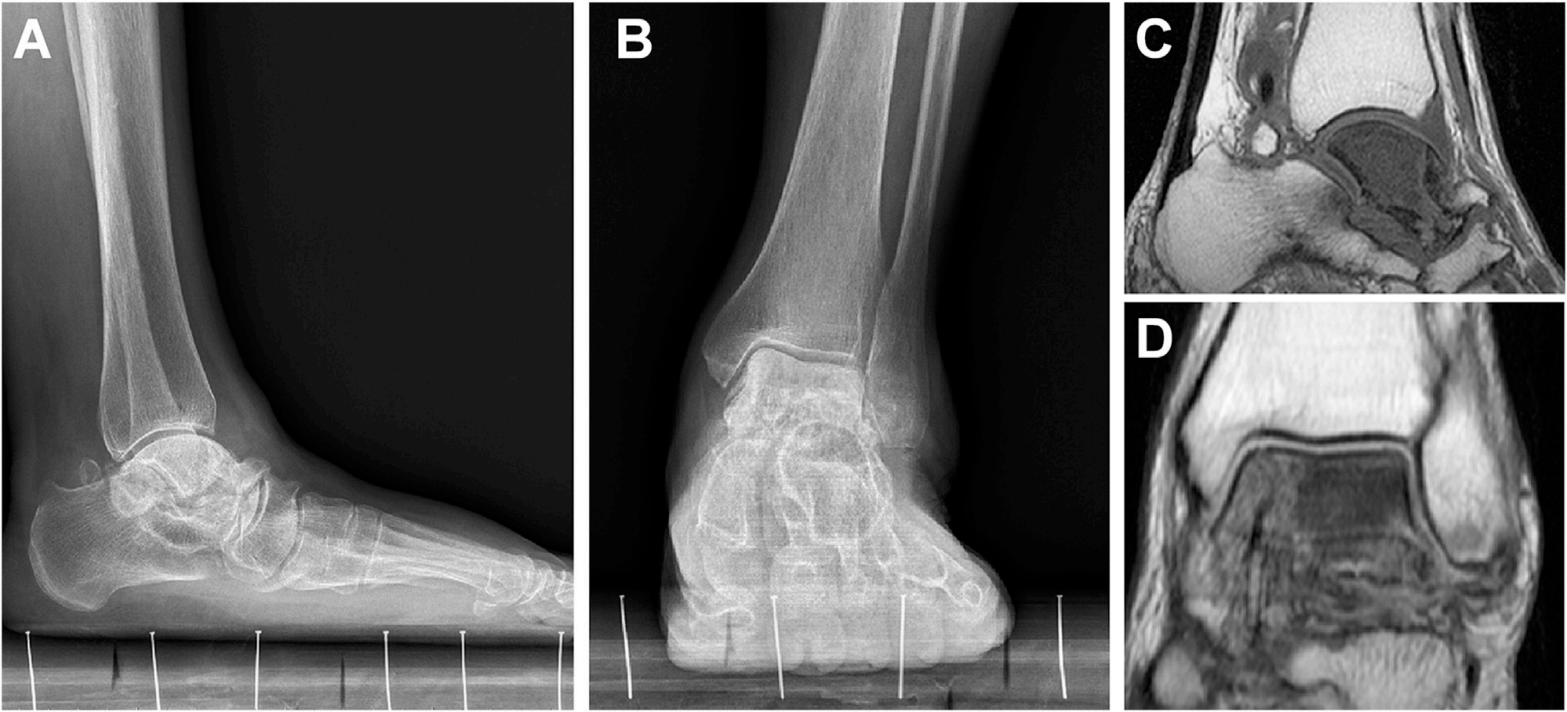
Preoperative weight-bearing anteroposterior and lateral views of patient 1 **(A,B)**. TAN was confirmed by magnetic resonance imaging **(C,D)**.

Between 2015 and 2019 three women aged 45–58 years with idiopathic talar necrosis underwent TTR with custom-made Vitallium prostheses designed by our team. Physical examinations, visual analogue scale (VAS) pain scores, and American Orthopedic Foot and Ankle Society (AOFAS) scores were recorded. Weight-bearing, anteroposterior, and lateral-view x-rays and magnetic resonance imaging were performed to confirm each diagnosis (Figure 1). CT scans of affected and unaffected sides were performed to facilitate the design and production of the prostheses. Patient characteristics are listed in Table 1.

**TABLE 1:** Patient details.

Patient no.	1	2	3
Age/gender	58/F	45/F	46/F
Involved side	L	L	bilateral
Duration of symptoms (months)	24	18	12
Diagnosis	talar necrosis	talar necrosis	talar necrosis
Hospital stays (days)	6	7	6
Duration of follow-up (months)	78	52	29
	Preoperative		
AOFAS score	35	59	37 (L), 61 (R)
VAS score	7	6	9 (L), 6 (R)
	Last follow-up		
AOFAS score	89	98	82 (L), 85 (R)
VAS score	3	0	6 (L), 1 (R)

### Data Acquisition and Image Processing

Bilateral ankle joint imaging data were acquired using a 64-slice spiral CT scanner (Philips Corporation, Japan; x-ray tube current 232 mA and KVP 120 kV, slice thickness 1 mm, reconstruction interval 1 mm).

### Prosthesis Design and 3D Printing Process

**FIGURE 2:**
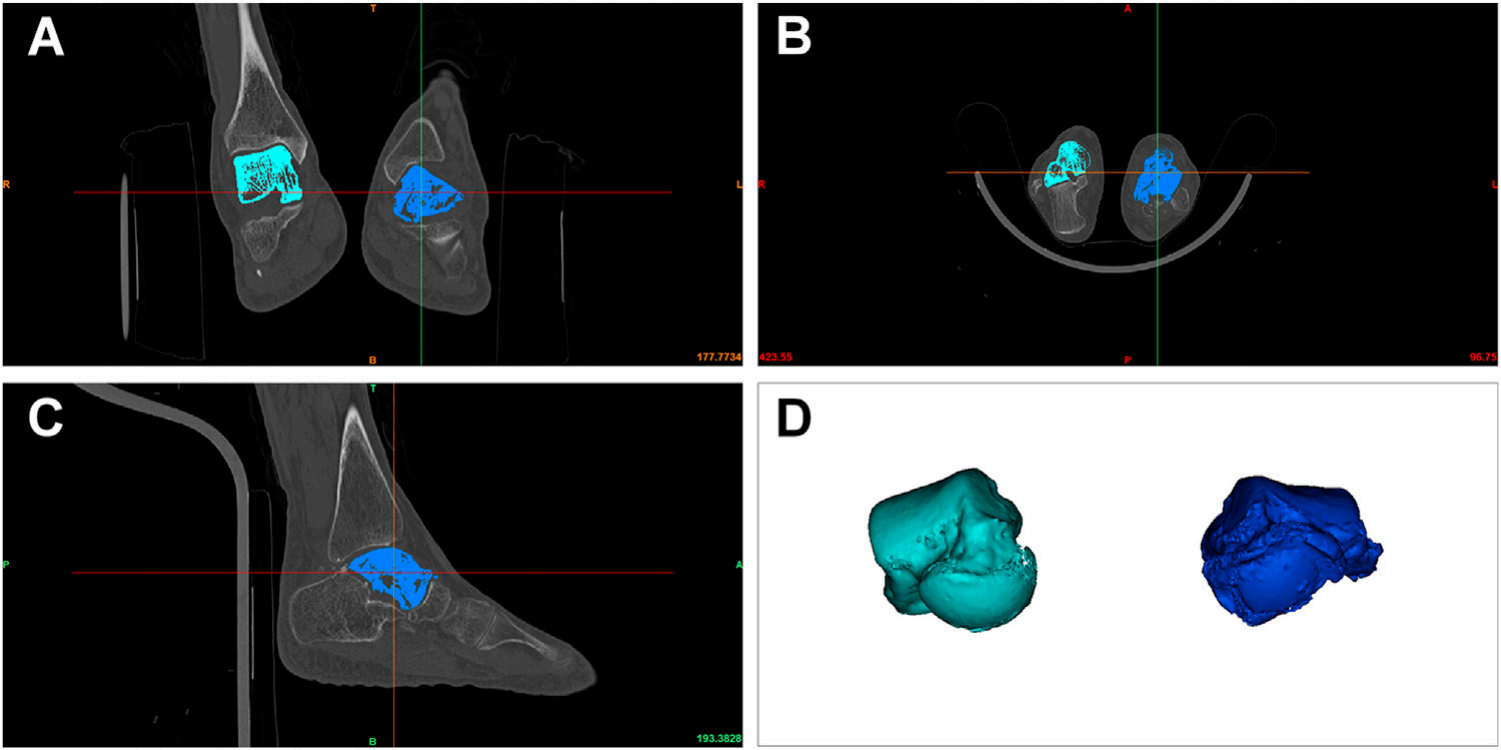
DICOM files obtained *via* CT were processed using Mimics software. Bilateral talars were located based on CT data **(A–C)**, and three-dimensionally reconstructed **(D)**.

**FIGURE 3:**
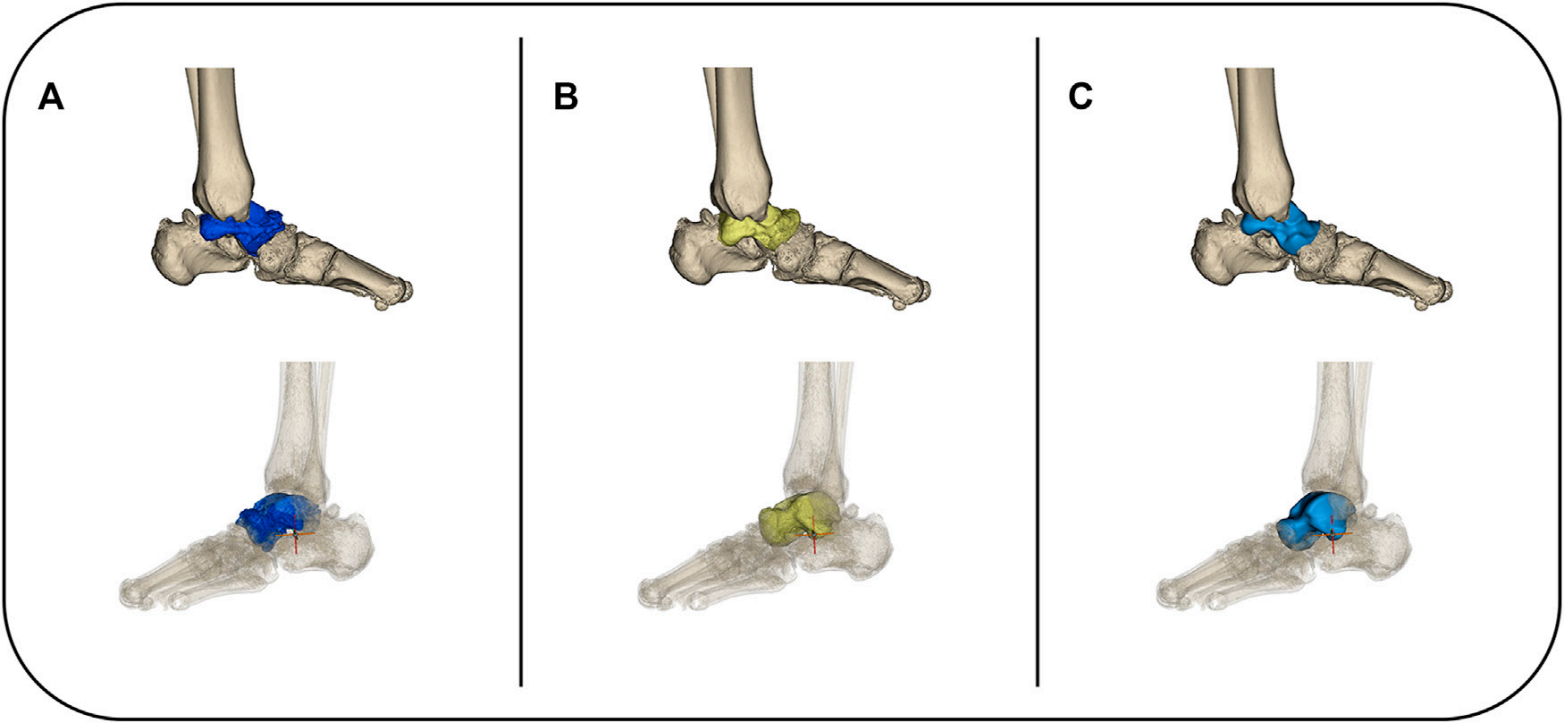
The affected talar was three-dimensionally reconstructed **(A)** and replaced using a mirror of the unaffected side *in situ* **(B)**. The reconstructed 3D model was then smoothed and wrapped until the overall and local details were satisfactory **(C)**.

**FIGURE 4:**
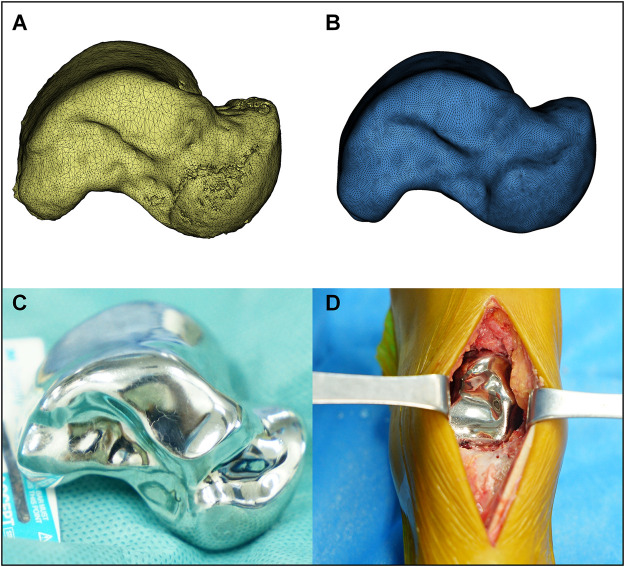
The mirrored model of the unaffected talar was finely trimmed **(A,B)** then 3D printed *via* electron beam melting technology. The prosthesis’ surfaces were then polished to reduce abrasion, and sterilized for clinical application **(C)**. TTR was performed in accordance with previously described studies **(D)**.

DICOM files obtained *via* CT scanning were acquired from all patients. Both the normal and the lesion sides of the talus were reconstructed using Mimics 17.0 software (Materialise Inc., Belgium). The normal side’s morphological features were considered the standard for the reconstruction of the talus prosthesis (Figure 2). For the patient with a bilaterally affected talus (case 3), according to previous research, the prosthesis design was based on the imaging data from the less distorted side to restore the initial shape of the talar. The 3D reconstructed model of the less affected talar was trimmed in CAD software, compared with the mirror images of both sides to recover the damaged lesion to the talar. (Taniguchi et al., 2012) The dimension parameters were readjustment according to the patient’s height and weight condition collected by our team. (Han et al., 2019) The reconstructed 3D model was then smoothed and wrapped using Mimics. The standard template library model was exported into Geomagic Studio 12.0 (Geomagic Inc., United States). The talus was smoothed repeatedly during this process *via* automatic and manual methods, until the overall and local details were satisfactory. The standard template library model was mirrored and compared with the talus of the lesion side. The size was rescaled several times until the implant model was consistent with the affected side (Figure 3). The prosthesis model was then exported to Magics 13.0 software (Materialise) to be sliced for printing. Vitallium alloy was used for 3D printing, which was conducted with the aid of Arcam EBM Q10plus. The printed prosthesis’ surfaces were then polished to reduce abrasion. Lastly the prosthesis was post-processed *via* ultrasonic cleaning, fine cleaning, and drying (Figure 4A–4C).

### Surgical Procedure

**FIGURE 5:**
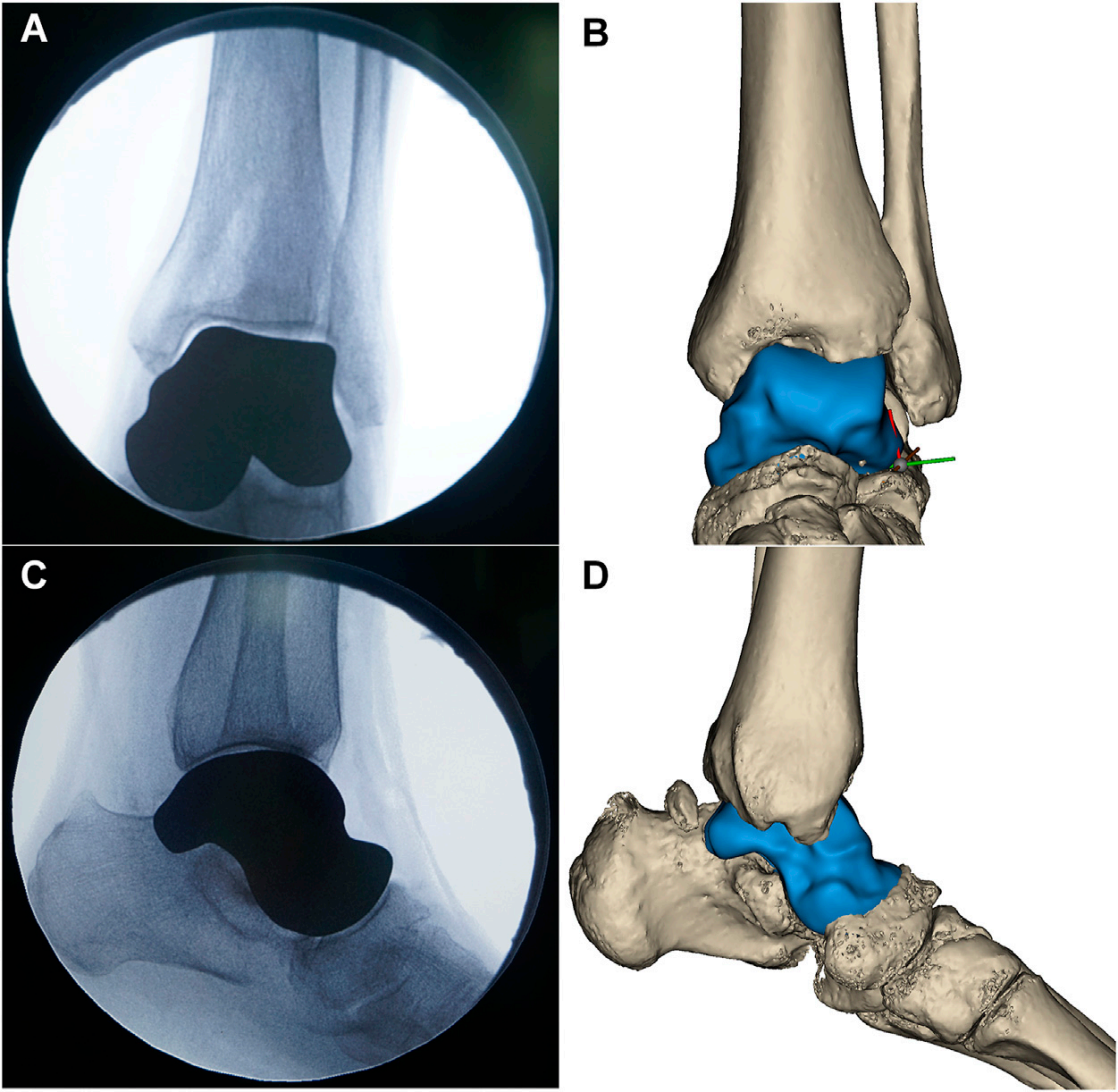
The position of the 3D printed talar prosthesis was confirmed by postoperative radiography **(A,C)**, and matched with the preoperative 3D reconstructed results **(B,D)**.

TTR was performed in accordance with previous studies. (Ando et al., 2016) An anteromedial incision was made between the tibialis anterior and extensor hallucis longus tendons. The articular capsule and ligament attachment of the talus were dissected, and the talus was extracted. After the talus was removed the calcaneus was retracted by hand, and the prosthesis was fixed inside. The implant stabilized between the tibia, calcaneus, and navicular, and exhibited no instability when the ankle joint was moved. The wound was sutured using absorbable sutures (#2-0). Ligament reconstruction was not performed (Figure 4D). Postoperative radiography confirmed the position of the 3D printed talar prosthesis (Figure 5). Each patient was allowed to walk and bear weight with the help of a walking boot after 1 week. To enable the surrounding soft tissues to heal, ankle joint exercise was not permitted until 4 weeks after surgery. All three patients could walk without an orthosis 4 weeks after surgery.

### Follow-Up

The patients were followed up at 6 months, 1 year, 3 years, and every 2 years after surgery. Weight-bearing, anteroposterior, and lateral view x-rays were taken to identify any signs of prosthesis loosening or degenerative change in the surrounding area of the joint. AOFAS and VAS scores, imaging, and videos of the patients in motion were recorded.

## Results

### Effects of Surgery

In all patients, the TTR operation was completed without any intraoperative complications. The 3D printed prostheses fit the original talar positions precisely and were enveloped steadily by ligaments and soft tissues (Table 1). There were no signs of wound infection, skin necrosis, or other surgical complications. Motion ranges and ankle joint stability determinations are shown in Table 1.

### Follow-Up Results

**FIGURE 6:**
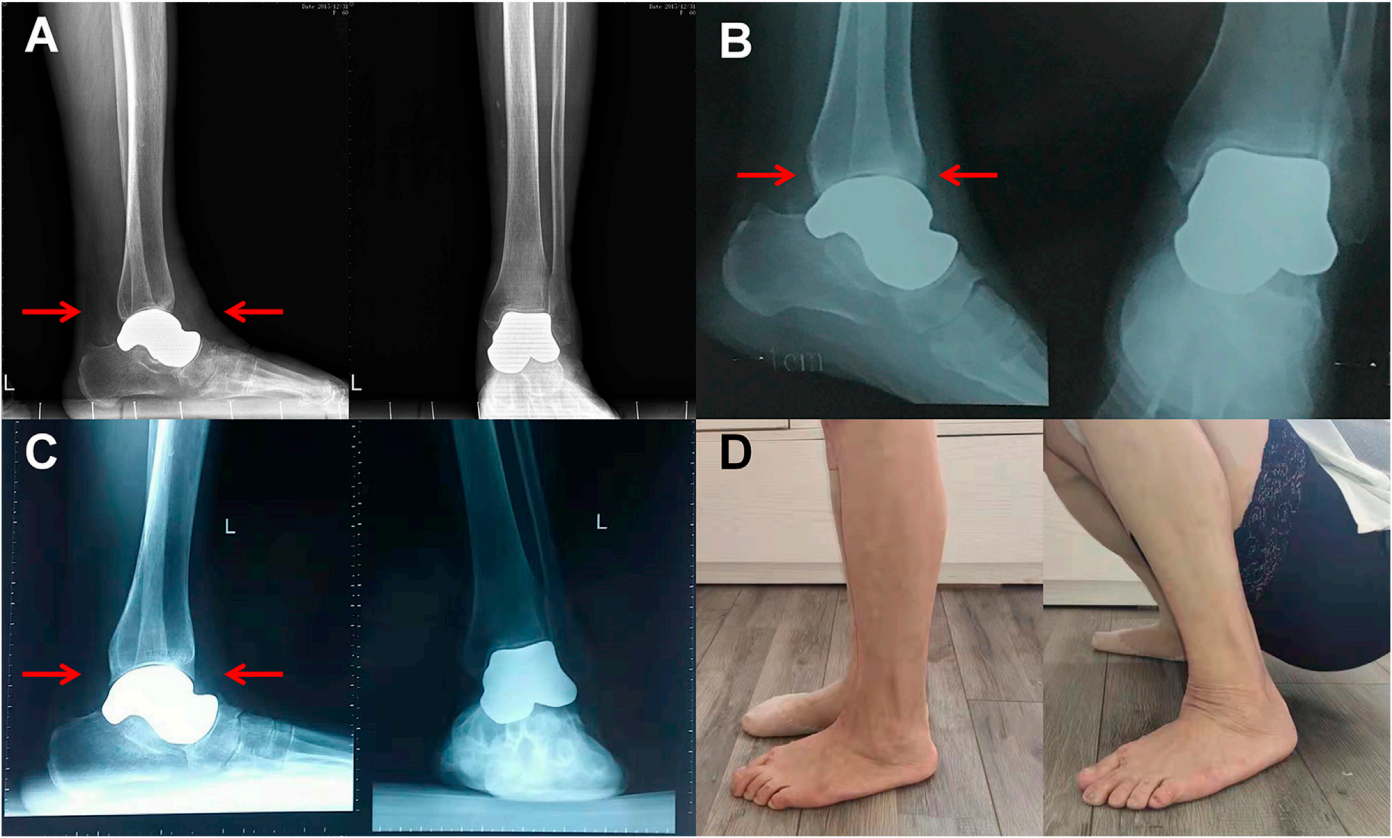
A 58 year-old woman (patient 1) suffered from pain and swelling of the left ankle for 2 years and was unable to walk without crutches. TTR with a 3D printed Vitallium talar prosthesis was performed in 2015. Anteroposterior and lateral radiographs taken postoperatively 1, 3, and 5 years after discharge did not depict any signs of prosthesis-related complications **(A–C)**. The formation of small osteophytes on the tibial side and navicular side was detected **(D)**, but the patient did not report any discomfort and had returned to daily activities.

The patients were followed up for 29–78 months. The most recent follow-up scores are shown in Table 1. In all patients there were no signs of prosthesis loosening or serious degenerative change in the surrounding area of the joint, but small osteophytes were observed on the tibial side and navicular side. No chronic infection or other prosthesis-related complications were observed in any of the patients after a mean follow-up of 53 months (Figure 6). The mean functional follow-up scores (AOFAS) increased from 48.0 to 88.5. The sagittal motion scores increased to normal or mild restriction in two patients, as did inversion and extension functions. In all three patients, pain scores had decreased markedly at the most recent follow-up. All patients were able to return to their daily lives without any walking aids. Patient 1 was a 58 year-old female patient who presented with a complaint of pain and swelling of the left ankle for 2 years. The patient was unable to walk without the help of crutches. Before the surgery, the patient exhibited limited left ankle movement, dorsiflexion was recorded to be 0°, while plantar flexion was 15°. The AOFAS and VAS scores were 35 and 7, respectively. In the last follow-up, the dorsiflexion was recorded to be 30° and plantar flexion was 20°. The patient reported moderate pain that occasionally occurred in the affected planta pedis, leading to comparatively poor follow-up scores. That patient was later diagnosed with plantar fasciitis and treated *via* physical therapy, and her most recent VAS score indicates slight improvement. Patient 2 was a 45 year-old female patient with swelling and pain in the left ankle with limited movement for 18 months. Before the surgery, the dorsiflexion and plantar flexion of the left ankle was recorded to be about 20°. The AOFAS and VAS scores were 59 and 6, respectively. In the last follow-up, the joint movement had shown noticeable improvement. The pain in the left ankle was also completely relieved. Patient 3 was a 46 year-old female who complained of bilateral ankle pain with movement limitation for more than 1 year. The left AOFAS and VAS scores were 37 and 9, and the right AOFAS and VAS scores were 61 and 6 before the operation. The dorsiflexion was about 15° on both sides and the plantar flexion was about 25° on both sides. Patient 3 complained of occasional pain in the left ankle while working. Physical examination revealed tingling and numbness occurred on the skin of the left ankle, which has led to an unsatisfied improvement in follow-up score. Indicating a potential injury of the cutaneous nerve. The patient refused our suggestion of neural block therapy. Meanwhile, the pain score in the most recent follow-up has slightly improved. Changes in this symptom remain to be observed.

## Discussion

Conservative treatment is not a viable option for terminal-stage TAN due to serious talar collapse and necrosis. Generally, small osteochondral lesions of the talus can be treated with core depression and bone grafting. All three patients in the current series had suffered severe damage to the whole articular surface and deformation of the talus however, therefore core depression and bone grafting were no longer suitable. Joint arthrodesis was also taken into consideration. The main drawbacks of ankle arthrodesis are stiffness and the effects of foot kinematics and ground reaction force progression of the foot, which can lead to degenerative osteoarthritis of the midtarsal joint due to pressure overload during walking. (Beyaert et al., 2004) Nonunion has been reported in approximately 10% of arthrodesis cases. (Haddad et al., 2007) Patient 1 would have had to receive tibio-talo-calcaneal arthrodesis, and the risk of nonunion would have been even greater. TAR was also an option to restore ankle function. Unfortunately, residual bone quality was relatively poor in all three patients, particularly with respect to potential progression of TAN, therefore there was a high associated risk of prosthesis loosening. In the present patients, TAR was only considered to be a salvage therapy at the present stage for patients with serious cartilage damage in the contralateral joint surface. While for patients with minor cartilage damage on the tibial plafond or calcaneal joint surface, reports of TTR did not consider this condition as a contraindication. (Taniguchi et al., 2015; Taniguchi and Tanaka, 2019; Yamamoto et al., 2021) This strategy was also seen in the studies of hemiarthroplasty of first metatarsophalangeal, in these cases, minor cartilage damages were commonly seen in the contralateral joint surface while the follow-up results noted excellent pain relief, high patient satisfaction, low reoperation rate, and no serious complications. (Butterworth and Ugrinich, 2019) Therefore, to avoid these limitations we opted for a custom-designed talar prosthesis, which provides multiple advantages such as negating specialized surgical techniques, reducing surgical trauma and postoperative complications, a relatively short recovery period, and preservation of joint function. At the most recent follow-ups, there were prominent improvements in joint function and quality of living, with no sign of prosthesis loosening or subsidence.

As a bony structure of the ankle joint, the talus is unique because it is devoid of any tendon or muscular attachments. The stability of the talus is maintained mainly by osseous and ligamentous structures. A thin cartilage profile measuring 1.0–1.7 mm covered 70% of the surface of the talus with extremely high compressive modulus and less elasticity, and was more prone to subchondral changes. (Gelberman and Mortensen, 1983; Burston et al., 2011) The cartilaginous interface of the talus between the tibial plafond, medial and lateral malleoli, calcaneus, and navicular aids in even distribution of weight-bearing forces on the ankle joint. (Rodgers, 1988) Together the cartilaginous interfaces withstand five times the weight of the body when walking, and up to 13 times the weight of the body when running, making the talus vital in the locomotive system but also rendering it vulnerable to trauma and subchondral changes. (Shnol and LaPorta, 2018) In a previous study talar prosthetic replacement exhibited a high long-term failure rate due to poor matching to patients’ individual talar anatomic and biological features. (Harnroongroj and Vanadurongwan, 1997) The talar prosthesis should have enough strength to brace the dynamic mechanical changes in the ankle joint, and be precisely matched to the complex morphological features of the cartilaginous interface. Studies indicate that a proper fit of the talus prosthesis correlates directly with long-term follow-up results. (Harnroongroj and Harnroongroj, 2014; Ando et al., 2016) Based on these principles, in the patients in the current series CT scanning of the unaffected side was used in conjunction with 3D printing methods to reconstruct the original characteristics of the talus. *Via* the 3D printing approach, the prosthesis could be conveniently rescaled and manufactured. Three sizes of talar prosthesis were prepared for our first patient—original size, plus 1 mm, and minus 1 mm—to simulate the thickness of the cartilage. The original sized prosthesis fitted the calcaneus best, without any signs of instability. With the aid of a 3D printed talus all patients achieved substantial increases in AOFAS and VAS scores. The prosthesis precisely replaced the necrotic talus, and stability was ensured by the surrounding tissues.

Given the complex biological environment and unique surficial features of the talus it is essential to make a talar prosthesis from a material with appropriate biological and biomechanical properties. The choice of prosthetic materials for TTR has evolved from bone cement (Tsukamoto et al., 2010) to alumina ceramic. (Taniguchi et al., 2012; Taniguchi et al., 2015; Taniguchi and Tanaka, 2019; Yamamoto et al., 2021) The primary aims during material selection are to improve the durability of the talar prosthesis and to reduce prosthetic wear. In the present cases, a metal material that has not been previously reported in the construction of talar prostheses was used, Vitallium alloy, and it exhibited satisfactory biocompatibility and minimal effects on corresponding bone and cartilage (Kyomoto et al., 2010; Dobruchowska et al., 2017) as well as low friction and high wear degradation resistance, which maximizes the durability of the service life of the prosthesis. (MacDonald and Wicker, 2016) The use of 3D printed Vitallium alloy has been reported in several previous studies.Chang et al. (2019) compared the marginal adaptation of 3D printed dental implants with that of traditionally fabricated implants. In that study, the 3D printed Vitallium alloy prosthesis met clinical requirements. Zheng et al. (2019) developed an innovative customized 3D printed total temporomandibular joint prosthesis constructed with Vitallium alloy that was safe and efficient in clinical use. When using this new material for TTR prosthetic design, we hoped the resulting custom-made prostheses would adjust the ankle joint, maintain stability, prevent degenerative changes in surrounding cartilaginous structures, and disperse complex dynamic pressure changes.

TTR has multiple advantages, including the preservation of a range of motion, limb length, and joint function, as well as the ease of the surgical technique and the short post-operative recovery period. Since it was first reported in 2010, (Tsukamoto et al., 2010) the fixation method in TTR has always been controversial. Theoretically there is a risk of instability if the talar prosthesis is dissociative in the ankle joint, because ligament reconstruction is not performed routinely in such surgery. (Ando et al., 2016) Hence, some surgeons preferred to fix the talar prosthesis to the calcaneus with screws to enhance stability. (Mu et al., 2021; West and Rush, 2021) Porous coating surfaces and other auxiliary devices have also been used to enhance prosthesis fixation in some studies. (Stevens et al., 2007; Regauer et al., 2017) Notably however, reported cases of prosthesis failure such as loosening and subsidence have mainly occurred at the bone-prosthesis interface. (Yamamoto et al., 2021) To eliminate interaction between the prosthesis and surrounding bone structure in the present cases, no screws or other bony fixation methods were used. The position of the talar prosthesis was restricted by the surrounding tissues. Benefiting from the advantages of 3D printing technology which offers a more precise and personalized way to fabricate talar prostheses. The surface of the prostheses would fit the surrounding joints’ facets more accurately to improve the joint’s stability. Moreover, to reduce the risk of instability, patients in this study were not allowed for weight-bearing exercise immediately after surgery. The patients were allowed to walk and bear weight with the help of a walking boot after 1 week. To ensure the surrounding soft tissue’s healing, ankle joint exercise was not permitted until 4 weeks after surgery. We believed that surrounding bones and residual ligaments and capsules could encapsulate the talar prosthesis and provide enough stability for daily activities. Radiography at the final follow-up 6.5 years postoperatively revealed small osteophytes on the tibial and navicular sides. It appeared to have been caused by the instability of the talar prosthesis. Nevertheless, the patient did not report any discomfort. Moreover, the osteophytes at the edges of the surrounding bones seemed to have improved the stability of the talar prosthesis, as the formation of the osteophytes had become diminished by the last follow-up. Radiographic follow-up focused on dynamic changes of the ankle joints is still needed. However, reconstructing ligament attachments on the prostheses has not yet been achieved, which is also a key point of our future research.

In terms of the advantages of manufacturing methods and material, the clinical application of the customized 3D printed Vitallium talar prosthesis described herein still has limitations. The number of patients is limited, and long-term follow-up with more detailed assessments is still required. Meanwhile, for now, we are only benefiting from the freedom and convenience of realizing our design provided by 3D printing methods. Further research by our team will focus on the mechanical analysis and structure characteristics of the 3D printed talar prosthesis, especially on the how 3D printing process affects the mechanical features of the material.

## Conclusion

In the current study, with the aid of 3D printing and a novel Vitallium alloy material, TTR achieved encouraging results in 3/3 patients. The patients were satisfied with their joint function, and were able to return to daily activities without limitation. Although more cases and longer-term follow-up still need to be studied, the success rate in the current series is encouraging.

## Data Availability

The original contributions presented in the study are included in the article/Supplementary Material, further inquiries can be directed to the corresponding authors.
